# Lignin triggers irreversible cellulase loss during pretreated lignocellulosic biomass saccharification

**DOI:** 10.1186/s13068-014-0175-x

**Published:** 2014-12-13

**Authors:** Dahai Gao, Carolyn Haarmeyer, Venkatesh Balan, Timothy A Whitehead, Bruce E Dale, Shishir PS Chundawat

**Affiliations:** Department of Chemical Engineering and Materials Science, Michigan State University, East Lansing, MI 48824 USA; Great Lakes Bioenergy Research Center (GLBRC), Michigan State University, 164 Food Safety and Toxicology Building, East Lansing, MI 48824 USA; Biomass Conversion Research Lab (BCRL), MBI Building, 3900 Collins Road, East Lansing, MI 48910 USA; Department of Biosystems and Agricultural Engineering, Michigan State University, East Lansing, MI 48824 USA; Department of Chemical & Biochemical Engineering, Rutgers, The State University of New Jersey, 98 Brett Road, Room C-150A, Piscataway, NJ 08854 USA

**Keywords:** Cellulosic biofuels, Cellulase adsorption, Enzymatic saccharification, Lignin, Non-specific enzyme binding

## Abstract

**Background:**

Non-productive binding of enzymes to lignin is thought to impede the saccharification efficiency of pretreated lignocellulosic biomass to fermentable sugars. Due to a lack of suitable analytical techniques that track binding of individual enzymes within complex protein mixtures and the difficulty in distinguishing the contribution of productive (binding to specific glycans) versus non-productive (binding to lignin) binding of cellulases to lignocellulose, there is currently a poor understanding of individual enzyme adsorption to lignin during the time course of pretreated biomass saccharification.

**Results:**

In this study, we have utilized an FPLC (fast protein liquid chromatography)-based methodology to quantify free *Trichoderma reesei* cellulases (namely CBH I, CBH II, and EG I) concentration within a complex hydrolyzate mixture during the varying time course of biomass saccharification. Three pretreated corn stover (CS) samples were included in this study: Ammonia Fiber Expansion^a^ (AFEX™-CS), dilute acid (DA-CS), and ionic liquid (IL-CS) pretreatments. The relative fraction of bound individual cellulases varied depending not only on the pretreated biomass type (and lignin abundance) but also on the type of cellulase. Acid pretreated biomass had the highest levels of non-recoverable cellulases, while ionic liquid pretreated biomass had the highest overall cellulase recovery. CBH II has the lowest thermal stability among the three *T. reesei* cellulases tested. By preparing recombinant family 1 carbohydrate binding module (CBM) fusion proteins, we have shown that family 1 CBMs are highly implicated in the non-productive binding of full-length *T. reesei* cellulases to lignin.

**Conclusions:**

Our findings aid in further understanding the complex mechanisms of non-productive binding of cellulases to pretreated lignocellulosic biomass. Developing optimized pretreatment processes with reduced or modified lignin content to minimize non-productive enzyme binding or engineering pretreatment-specific, low-lignin binding cellulases will improve enzyme specific activity, facilitate enzyme recycling, and thereby permit production of cheaper biofuels.

## Background

Biological-catalyzed transformation of cellulosic biomass to fuels, chemicals, and materials can address several imminent challenges faced by our society such as climate change, energy security, and rural economic development [1]. However, the transition from traditional crude oil to a biomass based economy is not simple. Lignocellulosic biomass recalcitrance to enzymatic and microbial-catalyzed deconstruction is one of the major factors hindering the production of inexpensive biofuels [2,3].

Enzymatic hydrolysis of lignocellulosic biomass requires a complex suite of hydrolytic and lytic enzymes (called CAZymes or carbohydrate-active enzymes; http://www.cazy.org) that synergistically depolymerize cell wall carbohydrate polymers into fermentable monomeric sugars [4]. Cellulose is the dominant carbohydrate polymer in cell walls and consists of several hundred β-1,4 linearly linked glucose polymer chains that aggregate into fibrils (24 to 36 chains) via hydrogen bonding and van der Waals interactions [5,6]. These cellulose fibrils can be depolymerized into soluble sugars by a combination of cellulolytic CAZymes, including endoglucanases (EGs), cellobiohydrolases (CBHs) or exoglucanases, and β-glucosidases (βGs) [2,7]. The classical mechanism for cellulose deconstruction has involved the synergistic action of EGs and CBHs, where EGs randomly hydrolyze internal glycosidic bonds in the cellulose chains while the CBHs processively attack the reducing or non-reducing ends of the cellulose chains. Binding of cellulases to the insoluble substrate is the essential first step towards deconstruction of cellulose to cellodextrins or glucose. CBHs and EGs typically contain a catalytic domain (CD) and a carbohydrate binding module (CBM) [2,8] joined by an extended interdomain linker peptide [9]. Previous work has suggested that the extent of binding of full-length cellulases to crystalline cellulose depends on both the CBM and the CD [10–12]. CBMs can enhance CD catalytic efficiency on insoluble substrates by increasing the local surface bound enzyme concentrations [8–10] and by targeting glycans specific to the CD [8,13].

In recent times, this classical endo-exo mechanism has been extended to include lytic polysaccharide monooxygenases that can oxidatively cleave cellulose glycosidic bonds instead of hydrolyzing them [4]. Nevertheless, EGs and CBHs remain the dominant CAZymes in commercial enzyme cocktails produced by industrial fungal strains (for example, *Trichoderma reesei*, now called *Hypocrea jecorina*) necessary to completely deconstruct pretreated cellulosic biomass into soluble sugars [14]. The minimum cocktail of *T. reesei* cellulases needed to hydrolyze cellulosic biomass includes EG I (Cel7B, [GenBank:M15665]; glycosyl hydrolase or GH family 7B), CBH I (Cel7A, [GenBank:CAH10320]; GH family 7A), and CBH II (Cel6A, [GenBank:M16190]; GH family 6A) [14–16]. Studies focused on optimizing the ratio of individual enzymes for pretreated biomass saccharification indicate that these three cellulases are not only critical to hydrolysis efficiency but are also the most abundant enzymes in native secretomes and optimized cocktails (>60-70%, total protein weight basis) [15,17–19]. One of the crucial issues, however, is the high enzyme loading (>20-30 mg protein/g glucan) necessary for complete biomass saccharification. This is largely due to the high abundance of crystalline cellulose fibrils [20,21], which have poor enzyme accessibility [22] by virtue of being embedded in an amorphous matrix of hemicellulose and lignin within the cell wall [6,23].

Lignin is thought to impede the activity of CAZymes in part because of non-productive binding of enzymes to its surface and/or through steric hindrance due to the lignin-carbohydrate complexes that decrease cellulose accessibility [22,24–33]. In addition, lignin-derived degradation products produced during pretreatment could further inhibit enzyme activity [34–36]. Recalcitrance to enzymatic saccharification is dependent on the total amount and type of lignin present within plant cell walls [37–39]. Consequently, delignification of biomass after pretreatment can enhance hydrolysis rate and overall sugar yield [33,40]. Several studies have reported on the beneficial effects of surfactants and other sacrificial proteins (like bovine serum albumin, or BSA) that likely prevent non-productive binding of cellulases to lignin to some extent [41–43]. However, the affinity of individual cellulases towards lignin is still far from clear. This is partly due to the lack of suitable techniques to track binding of individual enzymes within complex protein mixtures during biomass saccharification. Secondly, it is difficult to distinguish the contribution of productive (binding to specific glycans) versus non-productive (binding to lignin) binding of cellulases to complex lignocellulosic substrates. Conducting assays on purified cell wall components like acid-insoluble Klason lignin or pure cellulose (like Avicel™) to mimic individual components of pretreated biomass is one way to address this problem [24,44]. However, the major limitation of this approach is that the molecular and ultrastructure of lignin is modified during the isolation process, and hence its affinity toward enzymes may not be representative of native cell wall lignin. The pretreated cell wall ultrastructure (for example, lignin relocalization during pretreatment) and chemical linkages between different cell wall components (cellulose, hemicelluloses, and lignin) also cannot be simulated by physically recombining individually purified components. Furthermore, previous approaches to track binding of cellulases to insoluble biomass during saccharification have been conducted by measuring either total crude protein concentration, individual protein concentration by SDS-PAGE, and/or activities of unbound enzymes in the hydrolyzate using individual CAZyme specific substrates [32,33,44]. Such approaches have several limitations that have been highlighted previously [45].

To achieve a comprehensive understanding of cellulase binding to pretreated lignocellulosic biomass during saccharification, we have utilized an FPLC-based methodology to quantify the CBH I, CBH II, and EG I free enzyme concentration within a complex hydrolyzate mixture [45,46]. Three pretreated corn stover samples were included in this study: Ammonia Fiber Expansion (AFEX-CS), dilute acid (DA-CS), and ionic liquid (IL-CS). These three pretreatment processes produce substrates with a range of residual lignin concentrations and therefore allow us to compare cellulase adsorption characteristics as a function of lignocellulose composition. We have made several interesting observations in the course of these studies: (a) The relative binding affinity of individual cellulases varied depending not only on the pretreated biomass type but also on the type of cellulase; (b) the total extent of non-productively bound cellulase to residual insoluble lignin depended on the pretreatment type and was directly correlated with lignin abundance; (c) the varying thermostability of *Trichoderma reesei* cellulases suggests that the composition of the enzymes active within the cocktail can change drastically during the course of biomass saccharification; and (d) by preparing recombinant CBM1 fusion proteins (tagged to green fluorescent protein, or GFP), we have shown that family 1 CBMs are highly implicated in non-productive binding of full-length cellulases to lignin. Overall, these findings would aid in the development of efficient, pretreatment-specific cellulase cocktails and economic enzyme recycling options to reduce the cost of biofuel production.

## Results

### AFEX, IL, and DA pretreated corn stover composition

The composition of corn stover pretreated by three leading pretreatment technologies being developed at the GLBRC (Great Lakes Bioenergy Research Center), BESC (BioEnergy Science Center), and JBEI (Joint BioEnergy Institute) is shown in Table 1. AFEX-CS has almost an identical composition to that of the original untreated biomass, which was approximately 34.4% glucan, 22.4% xylan, and 11% acid-insoluble lignin (on a dry weight basis). The DA-CS sample has the least amount of xylan (3.3%) and consequently has the highest glucan (60.6%) and lignin (32.9%) content. IL pretreatment selectively removes most of the lignin (2.7% residual lignin left behind) from the CS while leaving behind the glucan (46.9%) and xylan (29.8%). All subsequent biomass saccharification assays were performed at 1% glucan loading (dry weight basis) so that the maximum theoretical glucose yields and total enzyme needed (mg protein/g glucan) are identical. However, maximum xylose and acid-insoluble lignin concentrations are substrate-dependent as shown in Table 1.

**Table 1:** **Composition of various biomass substrates and maximum expected concentration of glucose, xylose, and lignin in pretreated biomass hydrolyzates (1% glucan loading basis) tested in this study**

**Substrates**	**Biomass composition**			**Maximum concentration (g/L)**		
	**Glucan**	**Xylan**	**Lignin**	**Glucose**	**Xylose**	**Lignin**
IL-CS	46.9%	29.8%	2.7%	11.11	7.22	0.58
AFEX-CS	34.6%	19.6%	11.0%	11.11	5.66	3.18
DA-CS	60.6%	3.3%	32.9%	11.11	0.62	5.43

Compositional data are on a dry weight basis. Lignin values reported here are for acid-insoluble Klason lignin only. All analyses were carried out in triplicate with mean values reported. Standard deviations in all cases were less than 10% of the reported mean values.

### Biomass saccharification yields and concentrations of free cellulases

**Figure 1:**
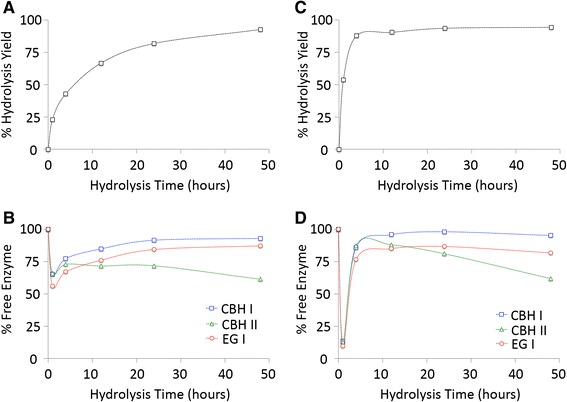
**Hydrolysis yield (A, C) of glucan to glucose and percentage of free cellulases in the hydrolyzate supernatant (B, D) for crystalline (A-B) and amorphous (C-D) cellulose.** Assays were carried out using a ternary cellulase cocktail of CBH I (blue squares), CBH II (green triangles), and EG I (red circles) for 48 h at 50°C. All assays were carried out in triplicate with mean values reported here. Standard deviations in all cases were less than 5%. Data for this figure have been reproduced from our previous study [46].

**Figure 2:**
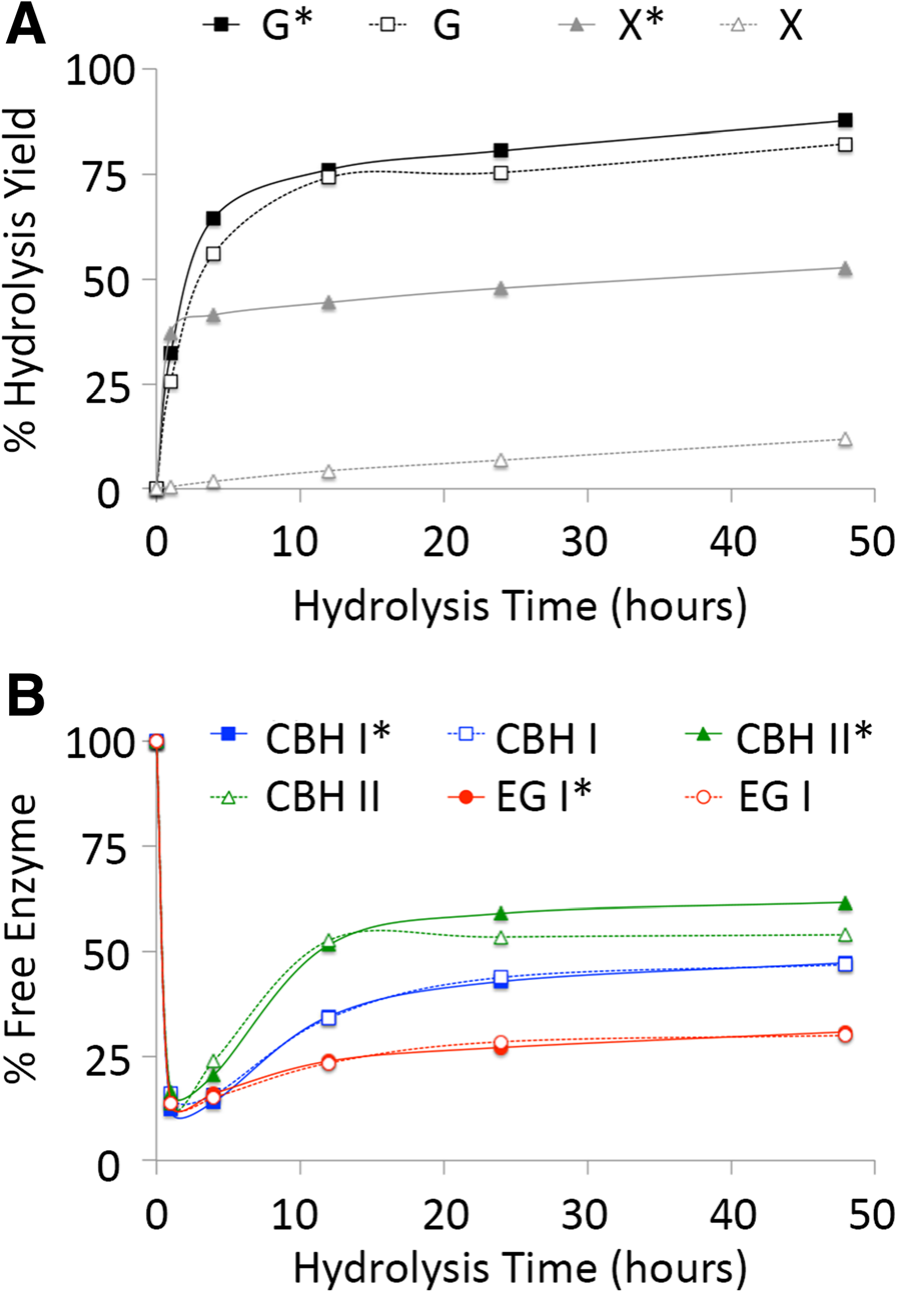
Hydrolysis yield (A) to glucose (G or G*) or xylose (X or X*) and percentage of free cellulases in the hydrolyzate supernatant (B) for IL-CS. Assays were carried out using a ternary cellulase cocktail of CBH I (blue squares), CBH II (green triangles), and EG I (red circles) either with (filled symbols with asterisk) or without (empty symbols with no asterisk) hemicellulase (endoxylanase and β-xylosidase) supplementation during the 48-h hydrolysis duration at 50°C. All assays were carried out in triplicate with mean values reported here. Standard deviations in all cases were less than 5%.

**Figure 3:**
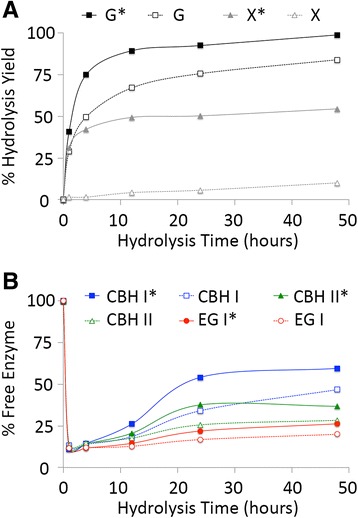
Hydrolysis yield (A) to glucose (G or G*) or xylose (X or X*) and percentage of free cellulases in the hydrolyzate supernatant (B) for AFEX-CS. Assays were carried out using a ternary cellulase cocktail of CBH I (blue squares), CBH II (green triangles), and EG I (red circles) either with (filled symbols with asterisk) or without (empty symbols with no asterisk) hemicellulase (endoxylanase and β-xylosidase) supplementation during the 48-h hydrolysis duration at 50°C. All assays were carried out in triplicate with mean values reported here. Standard deviations in all cases were less than 5%. Data for this figure have been reproduced from our previous study [46].

**Figure 4:**
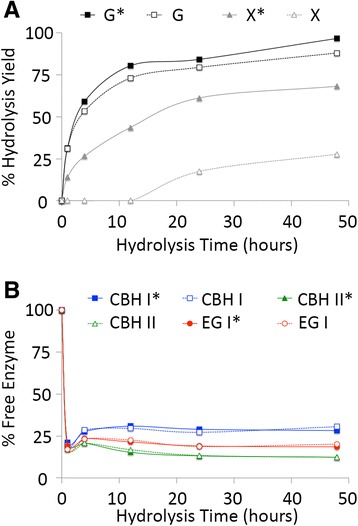
Hydrolysis yield (A) to glucose (G or G*) or xylose (X or X*) and percentage of free cellulases in the hydrolyzate supernatant (B) for DA-CS. Assays were carried out using a ternary cellulase cocktail of CBH I (blue squares), CBH II (green triangles), and EG I (red circles) either with (filled symbols with asterisk) or without (empty symbols with no asterisk) hemicellulase (endoxylanase and β-xylosidase) supplementation during the 48-h hydrolysis duration at 50°C. All assays were carried out in triplicate with mean values reported here. Standard deviations in all cases were less than 5%.

The saccharification yields and residual percentage of free cellulases bound to isolated cellulose (Avicel and amorphous cellulose), IL-CS, AFEX-CS, and DA-CS are shown in Figures 1, 2, 3, and 4, respectively. As shown previously [46], Avicel and amorphous cellulose gave greater than 90% glucose yield after 48 and 12 h, respectively (Figure 1). Since these substrates are predominantly cellulosic in composition (>99% on a dry weight basis), no additional assays were carried out in the presence of hemicellulases. More than 85% of CBH I, CBH II, and EG I were bound to amorphous cellulose within the first hour of saccharification. In contrast, a maximum of 35-45% of total added cellulases were bound to Avicel at an equivalent protein loading. Approximately 85-95% of the original added concentrations of all three cellulases were found in the hydrolyzate supernatant for amorphous cellulose after 12 h of saccharification. Similarly, close to 90% of added CBH I and EG I were found in the hydrolyzate supernatant for Avicel after 48 h of saccharification. Only about 60% of the initially added concentration of CBH II was recovered in the hydrolyzate for Avicel after 48 h.

IL-CS, which had the lowest lignin and highest xylan content among all pretreated CS substrates tested, gave close to 88% glucose yield and 53% xylose yield (Figure 2) after 48 h of saccharification by the ternary cellulase cocktail (CBH I, CBH II, and EG I) supplemented with hemicellulases (endoxylanase, EX, and β-xylosidase, βX). In the absence of supplemented hemicellulases, the ternary cellulase cocktail gave close to 82% glucose yield and 12% xylose yield after 48 h. Around 85% of the added cellulases were bound to the pretreated substrate within 1 h of saccharification. With increasing hydrolysis yields, the bound enzymes slowly returned to the supernatant at varying extents depending on the cellulase type. CBH II was the highest recovered cellulase with nearly 62% present in the hydrolyzate after 48 h (when supplemented with hemicellulases). Only 30% of EG I and 47% of CBH I were released back into the supernatant after 48 h, with little impact of hemicellulase addition on recovery of either cellulase.

AFEX-CS, which had intermediate lignin and xylan content among all the pretreated CS substrates tested, gave close to 84% glucose yield and 10% xylose yield (Figure 3) after 48 h of saccharification by the ternary cellulase cocktail. Supplementation of hemicellulases increased the glucose yield to 99% and the xylose yield to 55% after 48 h. The significant enhancement in both glucose and xylose yield for AFEX-CS upon addition of synergistic hemicellulases is consistent with our previous results [15,47]. As seen for IL-CS, most of the added cellulases (about 85-90%) are bound to the biomass after 1 h of saccharification. As the saccharification of residual cellulose and hemicellulose proceeded, a limited fraction of initially added cellulases were desorbed back into the hydrolyzate supernatant. The maximum fraction of CBH I (47% and 60% on added CBH I with and without hemicellulase supplementation, respectively) was found in the supernatant after 48 h of saccharification. For CBH II, a significantly lower fraction of added enzymes could be desorbed from the biomass. Hemicellulase supplementation increased CBH II recovery to about 37% after 48 h. For EG I, which had the poorest recovery among all three cellulases, only 20-26% of the total added amount could be detected in the supernatant with or without hemicellulase supplementation.

DA-CS, which had the highest lignin and lowest xylan content among all pretreated CS substrates tested in this study, gave close to 88% glucose yield and 28% xylose yield (Figure 4) after 48 h of saccharification by the ternary cellulase cocktail. Supplementation of hemicellulases increased the glucose yield to 97% and the xylose yield to 68% after 48 h. As seen previously [46], most of the added cellulases (about 80%) are bound to the biomass after 1 h of saccharification. However, unlike all other substrates, a significant fraction of added cellulases were still not recoverable for DA-CS (about 70-90%), even after 48 h of saccharification. A maximum of 30% of total added CBH I was detected in the supernatant, which is nearly half of the total recovered fraction for AFEX-CS. Approximately 20% of EG I and 12% of CBH II were detected in the DA-CS hydrolyzate supernatant, with minimal impact of hemicellulase addition on cellulase recovery.

### Relationship between cellulase recovery and total residual lignin concentration

**Figure 5:**
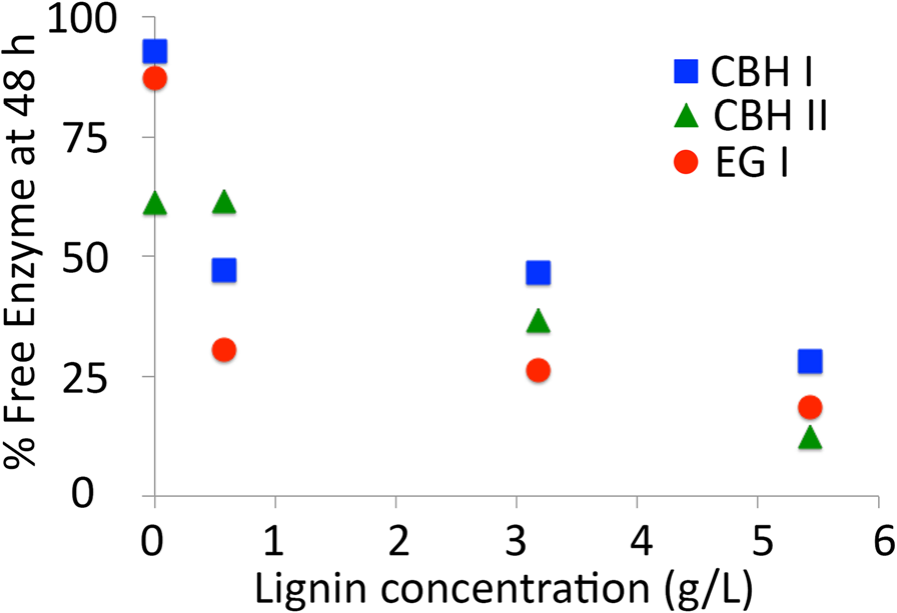
**Free cellulase concentration in the hydrolyzate supernatant as a function of total lignin concentration.** Data for free CBH I (blue squares), CBH II (green triangles), and EG I (red circles) after 48-h saccharification of Avicel (0 g/L lignin), IL-CS (0.58 g/L lignin), AFEX-CS (3.18 g/L lignin), and DA-CS (5.43 g/L lignin) at 50°C are reported here. All assays were carried out in triplicate with mean values shown here. Standard deviations in all cases were less than 5%.

The total fractions of free cellulases available in the hydrolyzate after 48 h of saccharification by a ternary cellulase cocktail for Avicel, IL-CS, AFEX-CS, and DA-CS are plotted against respective residual lignin concentrations in Figure 5. Fitting a linear regression trend line for CBH I (R^2^ = 0.63; slope = -0.0877; intersection = 0.74), CBH II (R^2^ = 0.99; slope = -0.0933; intersection = 0.64), and EG I (R^2^ = 0.53; slope = -0.0913; intersection = 0.62) indicates a marginal correlation between maximum recoverable cellulase fraction and total hydrolyzate lignin concentration. The y-axis intersection of these trend lines is not equal to unity, suggesting that the relationship between cellulase binding and lignin concentration is possibly non-linear. Other factors (such as cellulase thermal stability and lignin composition) may also play an important role in influencing cellulase recovery. The greatest drop in the recoverable fraction of cellulase with increasing lignin concentration (that is, the largest negative slope) was seen for CBH II > EG I > CBH I.

### Relationship between cellulase recovery and thermal denaturation

**Figure 6:**
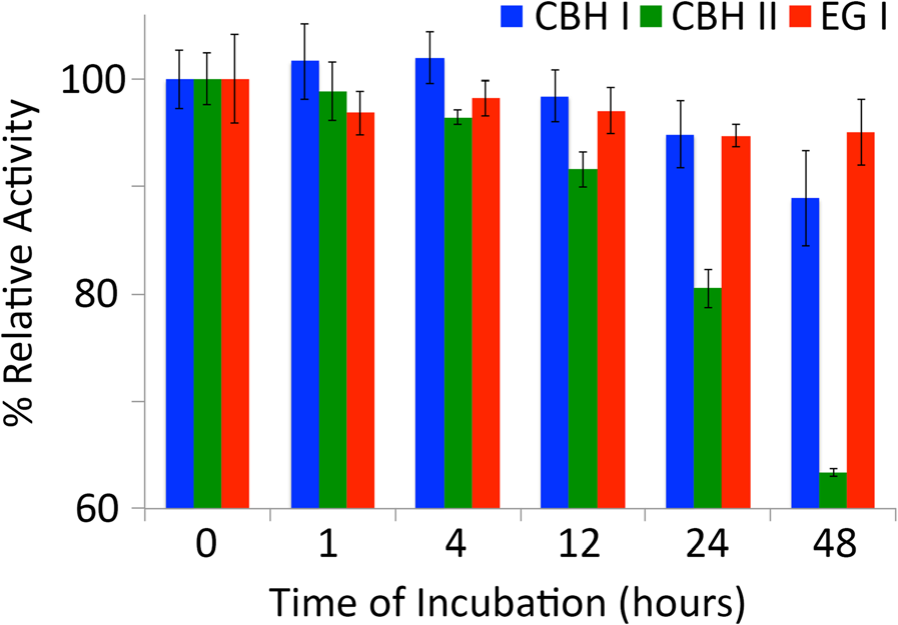
**Relative activity of CBH I (in blue), CBH II (in green), and EG I (in red) after incubation at 50°C for varying time periods.** Activity assays were conducted on CMC (for EG I) or Avicel (CBH I and CBH II). Error bars indicate standard deviations (±σ) for reported mean values. All assays were carried out in triplicate. Data for this figure have been reproduced from our previous study [46].

To determine the thermal stability of individual purified *T. reesei* cellulases, individual enzymes were incubated at 50°C in buffer alone for varying time periods. Periodically, samples were removed and assayed to determine specific activity (Figure 6), as shown previously [46]. After 48 h, a little over 60% of the original CBH II activity was retained. In contrast, CBH I and EG I retained more than 90-95% of their initial activity. A noticeable drop in cellulase activity is seen after 12 h of incubation at 50°C. These results clearly suggest that thermal denaturation of CBH II, as indicated by the loss in residual enzymatic activity, is likely responsible for the poor recovery during biomass saccharification as well.

### Role of family 1 CBM on binding full-length cellulases to lignin

**Figure 7:**
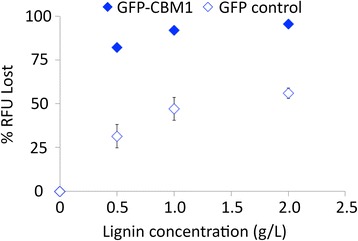
**Carbohydrate binding module (CBM) binds strongly to lignin isolated from AFEX-CS.** Here, percentage of protein fluorescence lost (% RFU Lost) due to GFP-CBM1 (filled blue diamonds) and GFP (empty blue diamonds) binding to lignin isolated from AFEX-CS is shown. For subtractive mass balance binding experiments, protein concentration was held constant at 0.2 μM and measured after 1 h of protein-lignin binding at 22°C. Error bars indicate standard deviations (±σ) for reported mean values and in some cases are smaller than the symbols. All assays were carried out in triplicate on two separate days.

In order to understand the role of family 1 CBMs in protein-lignin binding, CBM1 from *Trichoderma reesei* CBH I (Cel7A) was expressed as a genetic fusion to GFP (GFP-CBM1). GFP-CBM1 and a control GFP construct were prepared, and their relative binding to AFEX-CS derived lignin (>90% acid-insoluble Klason lignin; dry weight basis) was determined (Figure 7). In this assay, 0.2 μM of protein was incubated in the presence of different lignin concentrations at pH 6.0 in phosphate buffered saline. After 1 h of incubation, the amount of protein adsorbed onto lignin was recorded. At all concentrations, there is a significant difference in the binding of GFP-CBM1 versus the GFP control (as indicated by % relative fluorescence units, or RFU, lost in the supernatant) to lignin. At saturation, approximately 95% of the added GFP-CBM1 was bound to the insoluble lignin compared to 55% bound GFP control. These results suggest that the presence of a type A, family 1 CBM corresponds to an increase in protein-lignin binding, even at lignin concentrations as low as 0.5 g/L.

### Predicted hydrophobic patch scoring for cellulases using Rosetta

To gain a better understanding of the increased binding of CBMs to lignin and variations in the binding behavior for different full-length cellulases, we computationally determined the overall protein surface hydrophobicity for our experimentally tested constructs using the hydrophobic patch score in Rosetta [25]. This scoring term predicts significant enzyme-lignin binding for each of the three cellulases (Table 2). Previous work suggests a direct correlation between enzyme hydrophobic patch score and its binding to lignin [25]. The patch scores for full-length enzymes predict that EG I should have the lowest binding (Table 2), while CBH II should have the highest binding. Scoring the individual catalytic domains of each enzyme verifies the same rank order.

**Table 2:** **Hydrophobic patch score for full-length cellulases, individual catalytic domains (CDs), and carbohydrate binding modules (CBMs) for EG I, CBH I, and CBH II from** ***Trichoderma reesei***

	**Unweighted hydrophobic patch score**
Full-length EG I	6.8
Full-length CBH I	13.3
Full-length CBH II	27.9
EG I CD	6.2
CBH I CD	6.7
CBH II CD	23.7
EG I CBM	0.6
CBH I CBM	6.6
CBH II CBM	4.2

Structures were downloaded from the Protein Data Bank (PDB), modeled if necessary, and scored in Rosetta as described in the Methods section. Previous literature demonstrates that significant protein-lignin binding correlates with unweighted hydrophobic patch scores over 5 [25]. All full-length cellulases tested exceed this threshold.

## Discussion

By tracking the dynamic interaction of a minimal set of cellulases with various pretreated biomass substrates during saccharification, our experiments have revealed the relationship between enzyme recoverability and residual lignin concentration of the substrate. The current sets of experiments were carried out using a ternary cellulase cocktail (CBH I, CBH II, and EG I) with and without hemicellulase supplementation. Previous work has shown that the minimal ternary cellulase cocktail comprises close to 80% by mass of the total added enzymes necessary for complete cellulose saccharification [15,18,47]. However, for substrates like AFEX-CS and IL-CS, other auxiliary hemicellulases are necessary to achieve high hemicellulose hydrolysis yields [40].

For DA-CS and IL-CS, hemicellulase addition does not impact cellulase recovery. However, for AFEX-CS there is a significant improvement of cellulase recovery when hemicellulases are added. Because the gross hemicellulose composition of IL-CS is similar to AFEX-CS, it is possible that ultrastructural organizational differences in the xylan and cellulose might explain the improvement of cellulase recovery in the AFEX-CS case. AFEX pretreatment has been shown to remove some amount of hemicellulose along with alkali soluble lignin and redeposit it on the outer cell wall surface [23]. However, unlike for ILs, concentrated ammonium hydroxide used during AFEX does not swell or disrupt cellulose fibrils considerably to facilitate complete hemicellulose removal and precipitation [48]. A significant fraction of poorly accessible hemicellulose is interlaced between cellulose microfibrils [5,6] in native and AFEX treated cell walls [23]. It is therefore likely that this residual hemicellulose may be responsible for preventing complete cellulose saccharification and facilitates non-productive interactions with core cellulases. This could explain why the addition of hemicellulases that can hydrolyze and remove the residual hemicellulose has a greater impact on the recovery of core cellulases for AFEX-CS but not IL-CS. More research is clearly needed to better understand the impact of ILs on hemicellulose ultrastructure and lignin-hemicellulose complexation.

The net recoverability of the cellulase following 48 h of saccharification was closely dependent on the type of cellulase as well as the pretreatment chemistry. CBH I had the highest recovery for AFEX-CS and DA-CS, but surprisingly CBH II had the highest recovery for IL-CS after 48 h. This suggests that not only is the net lignin content influencing the total recovery of cellulases, but perhaps it is influencing the physical and chemical states of the lignocellulosic substrate as well. ILs are known to acetylate lignin, modify the syringyl-to-guaiacyl ratio, and decrease lignin ether content [49]. DA pretreatments can not only hydrolyze hemicellulose to soluble oligomers, but also result in cleavage of lignin ether linkages, acid-catalyzed lignin condensation, and formation of high surface area lignin nanoglobules [50]. AFEX pretreatment results in the cleavage of ester linkages with minimal modification of core lignin but can also form lignin-enriched globules [23]. Reducing the hydrophobicity of lignin by acetylation during IL pretreatment could explain the increased recoverability of CBH II, although more research is needed to rigorously test this hypothesis.

Although the rank order of cellulase-lignin binding predicted by hydrophobic patch score is not directly reflected in our experiments, all three cellulases contain significant hydrophobic patches predicted to impact enzyme-lignin binding [25]. However, the experimental results suggest that hydrophobic surface patches are not the sole contributing factor to lignin-mediated enzyme inactivation during hydrolysis. Previous work has shown that modification of lignin hydrophobicity can impact interaction of cellulases with lignin [25,29,51]. In the case of AFEX-CS and IL-CS, both exocellulases (CBH I and CBH II) had higher recovery after 48 h compared to EG I. We speculate that the increased non-productive interaction of endocellulases like EG I towards lignin could be due to the more open-cleft active site accessibility of aromatic amino acid residues that facilitates hydrophobic interaction with lignin [24]. More experimental evidence is needed to support this hypothesis. Previous research on optimization of the purified CAZyme cocktails for AFEX-CS has shown that EG I was required in greater proportions (nearly one-third of total enzyme added) than other enzymes for maximizing sugar yields [15]. Lower recovery of EG I versus exo-cellulases could explain why the optimal cocktail was skewed towards greater EG I content. These results indicate that non-productive interactions of enzymes with lignin (that lead to enzyme denaturation and likely irreversible precipitation due to lignin) can severely limit availability of free enzymes for efficient biomass saccharification. Previous work that focused on optimizing CAZyme cocktails to maximize pretreated biomass hydrolysis yields does not currently account for the relative stability of the enzymes tested in the presence or absence of lignin [15,17,47,52,53]. Our results suggest that, in addition to determining which CAZyme-specific activities are critical to maximizing the efficiency of the enzyme cocktail, it is also important to determine which enzymes are prone to denaturation and/or non-productive binding to residual lignin for each type of pretreated substrate.

*Trichoderma reesei* cellulases (namely exo-cellulase CBH II) are known to have poor thermal stability under process relevant conditions (at pH 5, 50°C, and with shaking) [54,55] and this finding was also confirmed in our recent work [46]. Thermally induced denaturation of exo-cellulases has been shown to be driven by aggregation and precipitation that resulted in reduced soluble protein concentration [56]. Binding of proteins to hydrophobic surfaces can further trigger conformational changes that drive enzyme denaturation as well [57]. This poor thermal stability is reflected in the lower net recoverable fraction of CBH II after completion of saccharification of Avicel and amorphous cellulose (hemicellulose and lignin are absent here unlike other substrates) compared with the other two cellulases. These results also suggest that drawing conclusions regarding reversibility of CAZymes binding to cellulose or lignocellulose based on free protein concentrations in the supernatant alone may have been premature, since protein precipitation due to poor thermal stability is likely unaccounted for in most depletion-based binding assays [24,58].

We have also shown that family 1 carbohydrate binding modules (CBM1 from CBH I) based GFP fusion proteins have higher relative affinity to AFEX-CS-derived acid-insoluble lignin compared to the GFP only control. Since all *Trichoderma* cellulase-derived CBMs belong to family 1 and are known to have a high pairwise sequence identity [59], we expect similar binding behavior for CBMs from CBH II and EG I to lignin. Previous work has suggested that CBMs and their hydrophobic aromatic amino acid residues facilitate non-productive cellulase binding to lignin in a pH-dependent manner [24,60]. Though the relative contribution of electrostatic versus hydrophobic interactions between cellulase-lignin cannot be clarified at this point in time [24,60], our results suggest that CBMs play an important role in triggering irreversible enzyme adsorption of full-length cellulases with residual lignin present within different pretreated substrates.

Overall, these findings allow a more detailed view of the mechanism of cellulase deactivation during lignocellulosic biomass saccharification and highlight the critical role of lignin towards facilitating this process. Decreasing cellulase cost in the overall production process centers on minimizing the role of lignin in promoting irreversible enzyme loss. From a pretreatment engineering standpoint, it would be critical to either remove lignin during pretreatment or chemically modify it to reduce its non-productive interaction with cellulases [26,61]. Alternatively, the cellulases could be engineered to minimize non-productive interactions with lignin while maintaining or improving specific activity and thermostability. Together, these efforts can synergistically reduce the high cost high of enzymatic saccharification for production of cellulosic fuels and chemicals.

## Conclusions

Here, we have determined the total recoverable core cellulases (CBH I, CBH II, and EG I) released into the hydrolyzate supernatant following pretreated biomass saccharification. The substrates tested here were pretreated by three of the leading pretreatment technologies (IL, DA, and AFEX pretreatments) currently under investigation by US Department of Energy (DOE) bioenergy research centers. Net recovery of core cellulases following complete saccharification of pretreated biomass was inversely related to the residual biomass lignin content. Acid pretreated biomass had the highest levels of non-recoverable cellulases, while IL pretreated biomass (with minimal lignin content) had the highest cellulase recovery, especially for thermally sensitive CBH II. Furthermore, non-productive interaction of CBM1 to residual lignin present within pretreated lignocellulosic biomass was implicated as a major factor triggering the poor recoverability of full-length core cellulases. Future work should focus on improving the recoverability of core cellulases to help increase enzyme specific activity and facilitate enzyme recycling for realistic lignocellulosic biorefinery substrates.

## Methods

### Crystalline and amorphous cellulose

Avicel (PH 101, Sigma-Aldrich, St. Louis, MO), which is predominantly cellulose in composition, was used to prepare amorphous cellulose using 83% phosphoric acid at 4°C for 60 min based on published protocols [21,46,62]. The cellulose crystallinity index for Avicel was estimated by the amorphous subtraction method, as described elsewhere [21], to be approximately 70%.

### AFEX pretreated corn stover (AFEX-CS)

A detailed protocol for preparing AFEX-CS has been provided previously [45,46] and is reproduced here. Milled CS was harvested in 2002 at Wray, Colorado (Pioneer Hybrid seed variety 33A14) and generously provided by the National Renewable Energy Laboratory (NREL, Golden, CO). The milled CS was AFEX pretreated at 60% moisture (kg water/kg dry biomass) and 1:1 ammonia (1 g ammonia/g dry biomass) loading at 130°C for 15 min total residence time. Details of the AFEX protocol and equipment used are provided elsewhere [34,63]. AFEX-CS, after air-drying in a hood overnight, was milled (Centrifugal mill ZM 200, Retsch, Newtown, PA) using a 0.08-mm sieve attachment as described previously [64]. The biomass composition (glucan, xylan, acid-insoluble lignin) was estimated based on the standard NREL laboratory analytical procedure (LAP) protocols (http://www.nrel.gov/biomass/analytical_procedures.html).

### Dilute acid pretreated corn stover (DA-CS)

A detailed protocol for preparing DA-CS has been provided previously [45] and is reproduced here. DA pretreatment was performed (at Professor Wyman’s lab at the University of California, Riverside) with a 1.0 L Parr reactor made of Hastelloy C (Parr Instruments, Moline, IL, USA). The CS was presoaked in 1.0% w/v dilute sulfuric acid solution at 5.0% solids (w/w) overnight. The total weight of the pretreatment mixture was 800 g. The presoaked slurry was transferred into the reactor, which was then sealed and fitted to the impeller driver motor, which was set at 150 rpm. The vessel was lowered into a hot sand bath and heated rapidly (within 2 min) to an internal temperature of 140 ± 2°C and maintained at 140 ± 2°C in the fluidized heating sand bath for 40 min. At the end of the reaction time, the reactor was cooled to below 50°C in a water bath. The diluted acid pretreated CS slurry was filtered through Whatman Grade Number 1 filter paper. Details of the apparatus, experimental procedure, and combined severity calculation are described elsewhere [65,66]. After pretreatment, the DA-CS residual solids were washed with water until neutral pH was achieved, dried at room temperature in a fume hood, milled using a 0.08-mm screen (as described above), and stored at 4°C. The composition of the DA-CS residual solids was estimated based on the standard NREL LAP protocol discussed above.

### Ionic liquid pretreated corn stover (IL-CS)

The IL pretreated CS was a generous gift from JBEI, and a detailed protocol for pretreatment is provided elsewhere [67]. Briefly, the CS was pretreated with 1-ethyl-3-methylimidazolium acetate at 140°C for 3 h at 10% (w/w) solids loading. Deionized water was added to the IL-biomass slurry to recover the dissolved polysaccharides, and the slurry was washed extensively to remove residual IL to recover the IL-CS. The composition of the IL-CS residual solids was estimated based on the standard NREL LAP protocol discussed above.

### Hydrolytic enzyme production and purification

Details of cellulase purification for CBH I, CBH II, and EG I are provided elsewhere [15]. Accellerase 1000™ from Genencor (Danisco US Inc., Genencor Division, Rochester, NY) was used to isolate CBH I, CBH II, and EG I. Details regarding production and purification of other enzymes used in this study (EX, βG, βX) are provided elsewhere [15]. The protein concentration was determined colorimetrically using the bicinchoninic acid or BCA protein assay kit (Pierce Biotechnology, Rockford, IL, USA) with bovine serum albumin (BSA) as the standard.

### Enzymatic hydrolysis

All hydrolysis experiments were performed in a 2.2-mL deep well microplate (Lot 780271, Greiner, Monroe, NC) at 1% (w/w) glucan loading along with 50 mM pH 4.5 citrate buffer in a total reaction volume of 500 μL. 15 mg/g glucan (corresponding to 0.15 mg/mL) each of CBH I, CBH II, and EG I were loaded along with 2 mg/g glucan of βG to prevent buildup of cellobiose. In addition, the ternary cellulase cocktails were supplemented with endoxylanase or EX (5 mg/g glucan) and beta-xylosidase or βX (2 mg/g glucan) to achieve near-theoretical glucan conversions within 48 h. The microplates were incubated at 50°C with shaking at 250 rpm for 48 h. Sampling was conducted at 1, 4, 12, 24, and 48 h. The supernatant was then separated from the insoluble solids by filtering through a 0.45-μm low protein binding hydrophilic microplate based filter (Lot R6PN00144, Millipore, Ireland) for protein and sugar analysis. All experiments were carried out in triplicate with mean values and standard deviations as reported in the figures. Glucose and xylose concentrations within the hydrolyzate were analyzed by HPLC as reported previously [34,46].

### Quantitation of free CBH I, CBH II, and EG I in hydrolyzate supernatant

The detailed methodology for individual cellulase quantification is published elsewhere [45,46] and is reproduced here. The difference in isoelectric points for CBH I, CBH II, and EG I allows them to differentially bind to an anion exchange column (Mono Q, Lot 17-5179-01, GE Healthcare) and elute out as individual components by applying a linear gradient of 1 M NaCl at pH 7.5. The concentration of individual enzymes was correlated to the elution peak area detected at UV 280 nm and calculated using the Unicorn 5.11 software. Before injecting the hydrolyzate (originally at pH 4.5) into the ion exchange column, a preliminary gel filtration step was applied to remove low molecular weight components that have UV absorbance as well as perform buffer exchange (to pH 7.5) [45]. Additional enzymes (βG, EX, and βX) were not quantified in this study. Since their molecular weights (βG and βX have Mw > 120 kDa and EX Mw < 25 kDa) are significantly different from those of CBH I, CBH II, and EG I (50 to 80 kDa), they do not interfere with the quantification of cellulases using this method [15]. The binding behaviors for βG, EX, and βX on a Mono Q column are different compared to CBH I, CBH II, and EG I. Hence, trace amounts of βG, EX, and βX in the cocktail do not affect the CBH I, CBH II, and EG I analysis. To reconfirm this, control experiments with and without βG, EX, and βX along with a cellulase cocktail (CBH I, CBH II, and EG I) were run, and no interference was found (data not shown).

### Thermal stability of CBH I, CBH II, and EG I

Detailed protocols for the thermal stability assays have been provided previously [46]. Briefly, 0.15 mg/mL of the individual cellulases were incubated at 50°C in buffer alone. Samples taken after varying incubation time periods were evaluated for cellulase specific activities on various substrates (CBH I and CBH II were tested on Avicel with incubation at 50°C for 24 h; EG I was tested on carboxymethyl cellulose with incubation at 50°C for 1 h). The reducing sugars were measured using a modified 3,5-dinitrosalicylic acid (DNS)-based assay [15]. Relative activities are reported based on samples from 0-h incubation.

### Lignin extraction

Lignin was extracted from milled AFEX-CS using established procedures [68]. Briefly, samples were extracted using a 90% (v/v) dioxane-nanopure water mixture with a solvent-to-biomass ratio of 20 mL solvent per g dry weight biomass. A metal heating mantle was used to hold this reaction at boiling under nitrogen atmosphere for 1 h. Product was filtered and neutralized before being concentrated to 20 mL using a rotary evaporator (Buchi, New Castle, DE, USA). To precipitate the lignin, the concentrated product was precipitated into 200 mL of stirring water. To remove residual dioxane, the lignin precipitate was washed extensively using pH 6.0 PBS buffer as follows: the precipitate was suspended in a tenfold volume of buffer and centrifuged, and the supernatant was decanted. Ten washing steps were performed for each extracted lignin sample. The composition of the extracted lignin was determined using the NREL LAP compositional analysis protocol (http://www.nrel.gov/biomass/analytical_procedures.html). Approximately 10% of the total biomass was extracted as purified lignin.

### Production of GFP-CBM and GFP protein constructs

A detailed map and protocols used for creating the generic pEC GFP_CBM plasmid are described elsewhere [69]. To generate the GFP_CBM1 construct, a family 1 CBM (from CBH I or Cel7A) was linked together via a 40 amino acid linker to GFP on the C-terminus and a His_8_ tag on the N-terminus. The CBM1 gene was synthesized by Genscript (Piscataway, NJ) and was inserted into an in-house pEC GFP_CBM vector using AflII and BamHI restriction enzymes, as documented elsewhere [69]. The plasmid was transformed into Rosetta-gami 2 [DE3] competent cells (Novagen, Santa Clara, CA) and inoculated into 50 mL non-inducing medium [70] with 50 μg/mL kanamycin and 25 μg/mL chloramphenicol. The culture was incubated overnight at 25°C and was used to inoculate 2 L of auto-induction medium. Cells were grown at 25°C for 27 h. Cells were harvested using centrifugation, and the cell pellets were stored at -80°C.

The cell pellets were thawed and resuspended in 50 mM Tris-HCl buffer with 50 mM NaCl and 15 mM imidazole (pH 7.4) with 0.5 μL/mL DNAse, 0.5 μL/mL lysozyme, and 1 μL/mL protease inhibitor. Cells were sonicated with an ultrasound sonicator (550 Sonic Dismembrator, Fisher Scientific, Pittsburgh, PA) fitted with a 1-inch probe at 4°C for 5 min with 30-s on bursts and 30-s off bursts. The cell debris was centrifuged at 14,000 rpm at 4°C (20 min) and the supernatant was collected for purification. The supernatant was purified using Ni-affinity column-based purification on an FPLC system (GE Healthcare, Pittsburgh, PA) as described previously [69]. While the GFP control protein did not require additional purification following the Ni-affinity step, the full-length GFP-CBM1 did require additional cleanup as discussed below.

To separate cleaved GFP subunits from the intact full-length GFP-CBM protein, a modified cellulose affinity purification step was employed [71,72]. Briefly, phosphoric acid swollen cellulose (PASC) or amorphous cellulose was produced according to previously outlined protocols [21,46,62]. The Ni-affinity purified crude protein mixture was applied to PASC at a ratio of 200 mg protein per g PASC (dry weight basis). This was incubated at room temperature with 150 rpm shaking. The sample was centrifuged at 4,000 rpm for 20 min at 4°C and the supernatant was discarded. One part pellet was resuspended with four parts (volume basis) 1 M NaCl, 10 mM MES, pH 6.0 and centrifuged again. One part pellet was resuspended with four parts (volume basis) ethylene glycol at room temperature with 150 rpm shaking and was centrifuged again. The supernatant was collected and was concentrated on the FPLC using a Ni-affinity purification column, as documented above. The eluted protein was desalted into pH 6.0 PBS. Protein concentrations were determined by measuring A_448_ of the chromophore using the NaOH denaturation method [73]. All chemicals were purchased from Sigma-Aldrich (St. Louis, MO), unless stated otherwise.

### Protein-lignin binding assay

A fluorescence-based subtractive mass balance assay was used to characterize proteins for lignin binding. Fluorescent proteins were assayed for lignin binding with dioxane-extracted AFEX-CS lignin of 95% purity. This assay was performed in 1-mL deep well plates (Lot 1896-1000, USA Scientific, Orlando, FL) sealed with thermoplastic elastomer capmats (Lot 1775-3083, USA Scientific, Orlando, FL). The 400-μL reaction volume comprised purified protein and extracted lignin, both in pH 6.0 PBS. The protein concentration was held constant at 0.2 μM in the reaction for all lignin concentrations tested (0 mg/mL, 0.5 mg/mL, 1.0 mg/mL, and 2.0 mg/mL). Samples were incubated for 1 h at 22°C. To ensure mixing between protein and lignin, end-over-end mixing via a bench top tube rotator (Argos Technologies, Elgin, IL) was used. Protein stability was accounted for by including unshaken samples at 4°C and 22°C and was also used to determine expected initial concentration. After 1 h of incubation, the samples were centrifuged and their supernatant collected. The fluorescence of the supernatant was measured using the Synergy H1 Hybrid Multi-Mode Microplate Reader from BioTek, Winooski, VT. Reader conditions were as follows: 481 nm excitation, 509 nm emission, gain of 50, and read height of 7.0 mm. The unshaken controls were used to estimate the fraction of lignin bound proteins for all samples. Comparing supernatant fluorescence to the signal in the 0 mg/mL lignin loading condition gives the fraction of protein lost due to lignin binding. Experiments were carried out in triplicate on two separate days.

### Hydrophobic patch scoring

Individual domains and full-length *T. reesei* cellulase structures were scored using the unweighted hydrophobic patch score term in Rosetta, as previously developed [25,74]. Biologically relevant versions of cellulase catalytic domains for EG I, CBH I, and CBH II from *T. reesei* were found in the Protein Data Bank [PDB:1eg1, 1cel, and 1hgw, respectively]. CBMs for CBH I [PDB:1cbh] and EG I [PDB:4bmf] were scored from previously solved structures. The structure for CBH II CBM was generated from the CBH I scaffold using the mutagenesis wizard in PyMOL [75] with the following mutations: T1C, Q2S, H4V, Y5W, I11Q, G12N, Y13W, V18C, T23S, Q26V, V27Y, L28S, P30D. All structures were cleaned and renumbered before the analysis in Rosetta. Validation of the hydrophobic patch scoring term with the results of Sammond and co-workers [25] was done by rescoring BSA.

## Endnote

^a^AFEX is a trademark of MBI, Lansing (http://www.mbi.org).

## Abbreviations

AFEX: Ammonia Fiber Expansion
AFEX-CS: AFEX treated corn stover
BESC: BioEnergy Science Center
BSA: bovine serum albumin
CAZymes: carbohydrate-active enzymes
CBH: cellobiohydrolase
CBM: carbohydrate binding module
CD: catalytic domain
CS: corn stover
DA-CS: dilute acid treated corn stover
EG: endoglucanase
FPLC: fast performance liquid chromatography
GFP: green fluorescent protein
GLBRC: Great Lakes Bioenergy Research Center
IL-CS: ionic liquid treated corn stover
JBEI: Joint BioEnergy Institute
PASC: phosphoric acid swollen cellulose
PDB: Protein Data Bank
RFU: relative fluorescence units
